# Anti‐inflammatory effects of reticuline on the JAK2/STAT3/SOCS3 and p38 MAPK/NF‐κB signaling pathway in a mouse model of obesity‐associated asthma

**DOI:** 10.1111/crj.13729

**Published:** 2024-01-19

**Authors:** Xiaojiang Lyu, Jiaojiao Liu, Zengrong Liu, Ying Wu, Ping Zhu, Chonghai Liu

**Affiliations:** ^1^ Department of Pediatrics Affiliated Hospital of North Sichuan Medical College Nanchong China

**Keywords:** asthma, inflammation, JAK/STAT, neutrophil, obesity, SOCS3

## Abstract

**Background:**

Asthma associated with obesity is a chronic disease characterized by earlier airway remodeling, severe wheezing, and increased insensitivity to hormone therapy. Reticuline, a bioactive compound of *Magnoliae Flos*, exerts anti‐inflammatory activity and can inhibit neutrophil recruitment. Thus, this study investigated the role of reticuline in obesity‐related asthma.

**Methods:**

The BALB/c mice fed a low‐fat diet (LFD) and high‐fat diet (HFD) were intranasally challenged with house dust mites (HDMs) or ovalbumin (OVA). Reticuline (0.25 mg/kg) was administrated into mice by intragastrical gavage. Airway hyper‐responsiveness was examined after the final challenge. Body weight was measured, and bronchoalveolar lavage fluid (BALF) and lung tissues were collected. The number of inflammatory cells in BALF was estimated. Histological changes were assessed by performing hematoxylin–eosin staining, and production of proinflammatory cytokines and IgE was examined by ELISA kits. Related pathways were studied with western blotting.

**Results:**

Reticuline suppressed airway resistance and inflammatory infiltration in lung tissue and reduced inflammatory cell recruitment in BALF in obesity mice with asthma. Additionally, the levels of IL‐17A, IL‐1β, IL‐5, macrophage inflammatory protein 2, and regulated on activation, normal T cell expressed and secreted in the lung were reduced by reticuline. Mechanistically, reticuline inactivated the JAK2/STAT3/SOCS3 and p38 MAPK/NF‐κB signaling pathways in obesity‐related asthma.

**Conclusion:**

Reticuline alleviates airway inflammation in obesity‐related asthma by inactivating the JAK2/STAT3/SOCS3 and p38 MAPK/NF‐κB signaling pathways.

## Graphical abstract

Reticuline suppressed airway resistance, inhibited inflammatory infiltration, and reduced the levels of inflammatory cytokines in obesity mice with asthma. Mechanistically, reticuline inactivated the JAK2/STAT3/SOCS3 and p38 MAPK/NF‐κB signaling pathways in obesity‐related asthma.

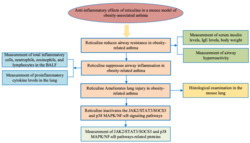

## 1 INTRODUCTION

Asthma is a chronic inflammatory disorder characterized by airway inflammation and hyperresponsiveness. Approximately 273 million people are afflicted with the disease worldwide, and there are estimated 495 100 deaths related with asthma.^1^, ^2^ Children are more vulnerable to asthma than adults, and 14% of children suffer from this chronic respiratory disease.^3^ Compared to adults, children with severe asthma have significantly higher number of eosinophils, allergen sensitizations, and higher IgE levels. Majority of children with asthma respond well to standard therapies; however, a significant proportion still have severe disease that is resistant to conventional therapies.^4^

Inhaled corticosteroids have been widely used to control asthma. However, approximately 10% of patients have severe asthma that is uncontrolled despite high doses of inhaled corticosteroids.^5^ Obesity increases asthma severity, and asthma incidence has elevated twofold in obese children.^6^ Obese asthma is typically associated with frequent exacerbations and low sensitivity to medical treatment.^7^ The pediatric obesity‐related asthma is multifactorial, including chronic inflammation, Th17‐induced neutrophilia, macrophage dysregulation, lipid metabolism, mitochondrial dysfunction, hormonal changes, insulin resistance, and body mechanics.^8^, ^9^, ^10^ As reported, severe asthma is associated with elevations in sputum neutrophils.^11^, ^12^ Clinical examinations of airway inflammation have been conducted in patients with severe obesity‐related asthma, and data suggest that obesity polarizes airway inflammation to a neutrophil‐dominant rather than eosinophil‐dominant phenotype.^13^, ^14^ Thus, better understanding the mechanisms of obese asthma pathogenesis is urgent, and attenuation of neutrophilic airway inflammation contributes to the treatment of obesity‐related asthma.

**FIGURE 1:**
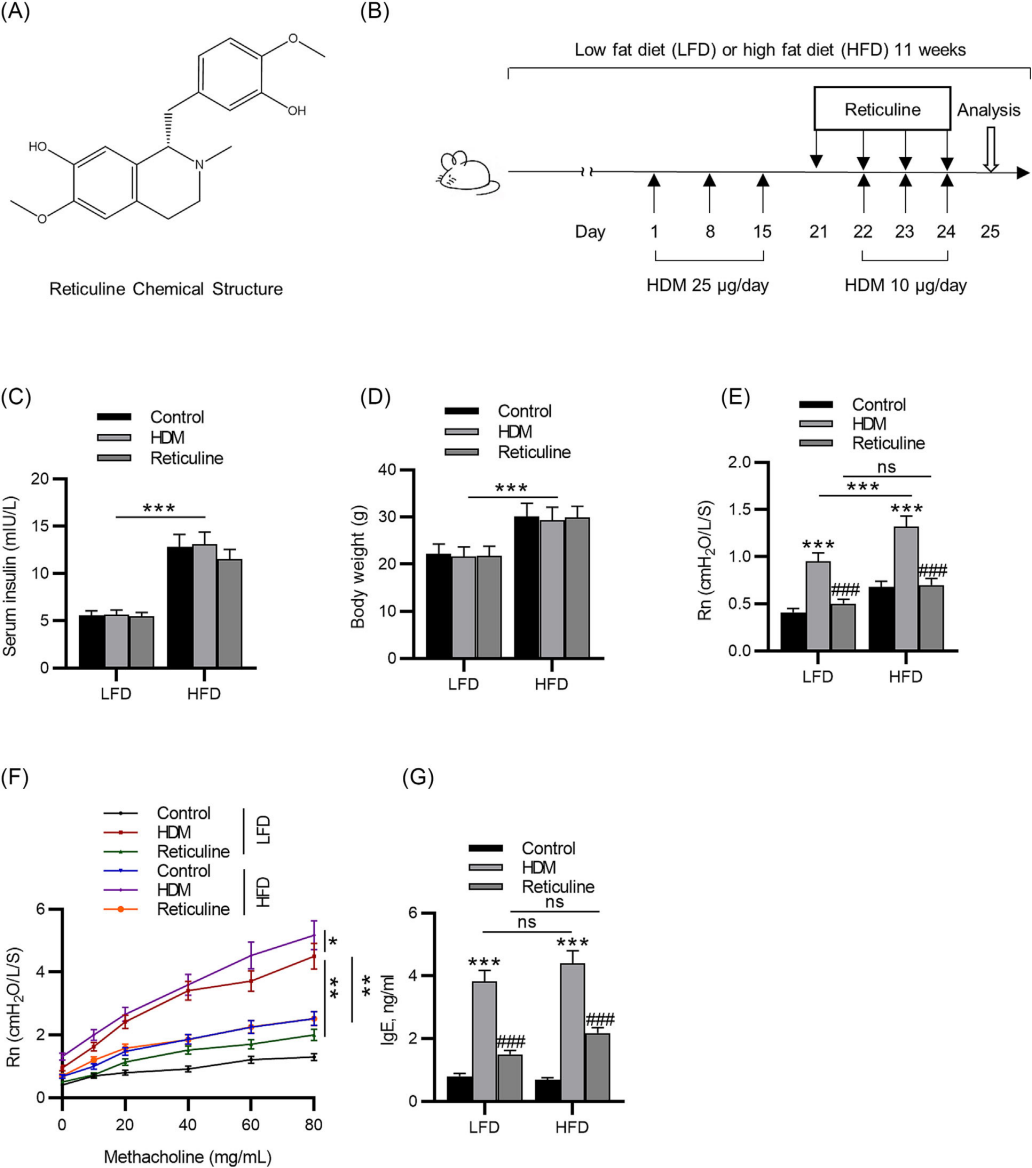
Reticuline reduces airway resistance in obesity‐related asthma. (A) Chemical structure of reticuline. (B) Protocol for HDM‐induced airway inflammation treated with reticuline in mice receiving LFD or HFD. (C) Serum insulin levels and (D) body weight after 11 weeks of LFD or HFD intake. (E) Baseline total airway resistance. (F) Methacholine‐challenged total airway resistance. (G) Serum IgE level measured by ELISA. *n* = 6 per group. ***p < 0.001 compared to control, ^###^ *p* < 0.001 compared to HDM. HDM, house dust mite; LFD, low‐fat diet; HFD, high‐fat diet.

Magnoliae Flos is widely used to treat asthma, sinusitis, allergic rhinitis, and headache.^15^, ^16^ Reticuline (Figure 1A) is a bioactive compound of Magnoliae Flos. It is the precursor to phenanthrene alkaloids, and the in vivo anti‐inflammatory assays suggest that reticuline has anti‐edematogenic potential and can inhibit neutrophil recruitment.^17^ The anti‐inflammatory activity of this substance has also been demonstrated.^18^ However, the biological functions of reticuline in obesity‐related asthma are still unknown.

The JAK/STAT pathway is involved in mediating cell proliferation, differentiation, apoptosis, and immune homeostasis, and is associated with the secretion of cytokines and growth factors.^19^ In recent years, this pathway has been reported to be implicated in obesity and metabolic syndrome.^20^ STAT phosphorylation can lead to activation of SOCS3 protein, which is involved in the pathogenesis of inflammatory disease.^21^ A previous study suggests that reticuline downregulates the level of JAK2 and STAT3 phosphorylation.^18^

House dust mites (HDM) are highly allergenic and exposure often associates with an urban sedentary indoor lifestyle, also resulting in obesity.^22^ The simultaneous exposure to low‐fat (LFD) or high‐fat diet (HFD) feeding and HDM extract have been widely used to establish the in vivo models of obesity‐related asthma.^23^, ^24^, ^25^ In this study, we investigated whether the effects of reticuline on obesity‐related asthma in HFD and HDM‐induced mouse models are mediated by the JAK/STAT signaling. We believe that this study would provide a theoretical basis for initiating novel promising therapeutic options for obese asthma.

## 2 METHODS

### 2.1 Animals

BALB/c mice (male, 6–8 weeks old, 20–25 g) were obtained from Charles River (Beijing, China), and housed at a constant room temperature of 22–25°C with three mice per cage under a 12‐h light/dark cycle. Animals were fed for 11 weeks either a low‐fat diet (LFD; D12450J; Research Diets Inc, New Brunswick, NJ, USA) or a high‐fat diet (HFD; D12492; Research Diets Inc). The LFD provided 10% of energy in the form of fat and the HFD provided 60%. Animal care and experimental protocols conformed to the Guide for the Care and Use of Laboratory Animals and were approved by Ethics Committee of Affiliated Hospital of North Sichuan Medical College.

### 2.2 Experimental model of asthma

For HDM‐induced obesity‐related asthma models, house dust mite (HDM) extracts of *Dermatophagoides farinae* were purchased from Institute of Tokyo Environment Allergy (ITEA, Tokyo, Japan). After 8 weeks of LFD or HFD intake, the mice were intranasally administrated with 25 μg HDMs on Days 1, 8, and 15, and 10 μg HDMs on Days 22, 23, and 24. Next, the mice were administrated with 0.25 mg/kg reticuline (purity: > 98%; ChemeGen, Shanghai, China) or a placebo (PBS containing 5% dimethyl sulfoxide) by intragastrical gavage on Days 21, 22, 23, and 24. The reticuline (0.25 mg/kg) was administrated 2 h before HDM administration on Days 22, 23, and 24 (Figure 1B). The dose for reticuline was determined according to the previous studies.^18^ The mice were divided into 6 groups: LFD‐PBS‐placebo (LFD‐control), LFD‐HDM‐placebo (LFD‐HDM), LFD‐HDM‐reticuline (LFD‐reticuline), HFD‐PBS‐placebo (HFD‐control), HFD‐HDM‐placebo (HFD‐HDM), and HFD‐HDM‐reticuline (HFD‐reticuline).

For OVA‐induced obesity‐related asthma models, the mice were divided into six groups: LFD‐PBS‐placebo (LFD‐control), LFD‐OVA‐placebo (LFD‐OVA), LFD‐OVA‐reticuline (LFD‐reticuline), HFD‐PBS‐placebo (HFD‐control), HFD‐OVA‐placebo (HFD‐HDM), and HFD‐OVA‐reticuline (HFD‐reticuline). As previously described,^26^ the mice were actively sensitized with an intraperitoneal injection that contained 10 μg ovalbumin (OVA, Sigma‐Aldrich, St. Louis, MO, USA) mixed with 20 mg Al (OH)_3_ gel in 0.1 mL normal saline on days 1 and 7 of the ninth week. From Days 18 to 25, the sensitized mice were challenged in a sealed container filled with 1% OVA aerosol (1 mg/mL) for 30 min once daily (Figure S1A). Aerosols were generated with an Inqua Neb PLUS (Pari, Starnberg, Germany) and delivered to the container using bias flow of medical air. The reticuline treatment groups received 0.25 mg/kg reticuline intragastrically at 48 h and 24 h before the first ovalbumin challenge.

### 2.3 Measurement of airway hyperreactivity

Total airway resistance was measured within 24 h after the final challenge as previously described.^27^ The mice were ventilated at a frequency of 150 breaths/min with a tidal volume of 0.2 mL and a positive end expiratory pressure of 2 cmH_2_O by a specialized ventilator (FlexiVent; SCIREQ, Montreal, Canada). Baseline airway resistance was measured after aerosolized saline. Then the mice were challenged by an increasing dose of aerosolized‐methacholine (Into Industrial Club in Tokyo, Japan) (10, 20, 40, 60, 80 mg/mL) every 3 min. Aerosols were generated with an ultrasound nebulizer and delivered to the inspiratory line of the FlexiVent.

### 2.4 Sample collection

The mice were intraperitoneally injected with pentobarbital sodium (100 mg/kg) and sacrificed after the last challenge. Body weight was evaluated, and then their bronchoalveolar lavage fluid (BALF) and lung tissues were collected. BALF was collected using the previous method.^28^ Briefly, a 23‐G tube was inserted into the trachea, and lung lavage was performed twice using PBS. The harvested BALF was subjected to a 5‐min centrifugation at 100 *g* at 4°C, and the supernatant was stored at −80°C.

### 2.5 Cell counts in BALF

After resuspending the cell pellet using 200 μL PBS, a hemocytometer (BioRad, Shanghai, China) was applied to count total cells in 50 μL cell suspension. Another 50 μL cell suspension was stained by Diff‐Quick (Solarbio, Beijing, China) to examine differential cell counts. The percentages of eosinophils, neutrophils, and lymphocytes in BALF were determined by counting 500 leukocytes in randomly selected fields under a light microscope.

### 2.6 Histological examination

The lung was fixed in 4% paraformaldehyde (Solarbio) for 24 h, embedded in paraffin, and cut into 4‐μm‐thick sections. The paraffin‐embedded sections were stained with hematoxylin–eosin (H&E), and peribronchial and perivascular infiltration was evaluated under a light microscope. Lung inflammation was assessed semi‐quantitatively as previously described^29^: 0 = absence of peribronchial inflammatory cells, 1 = a few peribronchial inflammatory cells infiltrate (25%), 2 = focal peribronchial inflammatory cells infiltrate (25%–75%), 3 = one definite layer of peribronchial inflammatory cells, 4 = two or more layers of peribronchial inflammatory cells. Slides were examined in a blinded fashion by three investigators. For each lung section, six randomly selected fields were scored, and mean peribronchial inflammatory score was calculated by adding the scores of all individual bronchioles and averaging the scores by the number of bronchioles present in the lung section. Neutrophils and eosinophils were counted (magnification: 400×) by two investigators in a double‐blinded manner, as described previously.^30^ The mean width of the observed area was 100 μm.

### 2.7 ELISA

The proinflammatory cytokine levels were measured in the lung homogenates. Briefly. the lung tissues were isolated and minced into small pieces. The tissues (10 mg) were homogenized in 100 μL cold PBS, and then the supernatants were collected after centrifuging at 1500 *g* for 15 min. The IL‐17A, MIP‐2, IL‐1β, IL‐13, IL‐5, and RANTES levels in the lung homogenates and the IgE levels in the serum were measured using ELISA kits (Solarbio) according to the manufacturer's instructions.

### 2.8 Western blotting

Lung tissues (50 mg per mouse) were homogenized in ice‐cold RIPA buffer (Sigma‐Aldrich) with a 1% protease inhibitor cocktail and a phosphatase inhibitor, and protein concentration was examined using a BCA Protein Assay Kit (Beyotime, Shanghai, China). Thereafter, proteins (40 μg) were separated by 10% SDS‐PAGE and blotted onto a PVDF membrane. After blocking with 5% skimmed milk for 2 h, the membrane was incubated overnight with primary antibodies against p‐JAK2 (ab32101, 1:4000; Abcam), JAK2 (ab108596, 1:5000; Abcam), phosphorylated STAT3 (ab76315, 1:5000; Abcam), STAT3 (ab68153, 1:2000; Abcam), SOCS3 (ab280884, 1:1000; Abcam), p‐NF‐κB p65 (ab76302, 1:1000; Abcam), NF‐κB p65 (ab32536, 1:1000; Abcam), p‐p38 MAPK (ab195049, 1:1000; Abcam), p38 MAPK (ab170099, 1:1000; Abcam), and GAPDH (ab9485, 1:2500; Abcam) at 4°C, and subsequently incubated with secondary antibodies for 2 h at room temperature. The bands were visualized by enhanced chemiluminescence reagent (Beyotime), and the intensity of blot was quantified by Image Lab 3.0 software (Bio‐Rad, Hercules, CA, USA).

### 2.9 Statistics analysis

All experiments were performed at least three independent repeats. Statistical analysis was analyzed using GraphPad Prism 8 (GraphPad Software, San Diego, CA, USA). Data are shown as the mean ± standard deviation. One‐way or two‐way analysis of variance followed by Tukey's post hoc analysis was used for comparison analyses. *p* < 0.05 was considered statistically significant.

## 3 RESULTS

### 3.1 Reticuline reduces airway resistance in obesity‐related asthma

After 11 weeks of an LFD or HFD intake, the effects of reticuline on serum insulin levels were investigated. The HFD mice had increased serum insulin levels compared with the LFD mice, suggesting HFD‐fed mice became diabetic and insulin‐resistant after 11‐week HFD diet. Treatment with reticuline did not affect serum insulin levels in mice (Figure 1C). The body weight of mice was measured, and the results showed that the HFD group exhibited a significantly greater body weight than the LFD group. Treatment with reticuline did not affect the body weight (Figure 1D). Baseline airway resistance of obesity‐related asthma mice was notably elevated compared to that of either obesity group or asthma group (Figure 1E). Airway resistance was gradually increased with an increasing concentration of aerosolized‐methacholine (Figure 1F). Additionally, airway resistance was high in obesity‐related asthma mice compared to asthma mice and it showed a significant improvement with reticuline treatment in obesity‐related asthma mice (Figure 1E,F). The IgE level was upregulated in the serum of asthma mice and obesity‐related asthma mice compared with control. Administration of reticuline remarkably downregulated this increase in both LFD and HFD groups (Figure 1G).

### 3.2 Reticuline suppresses airway inflammation in obesity‐related asthma

**FIGURE 2:**
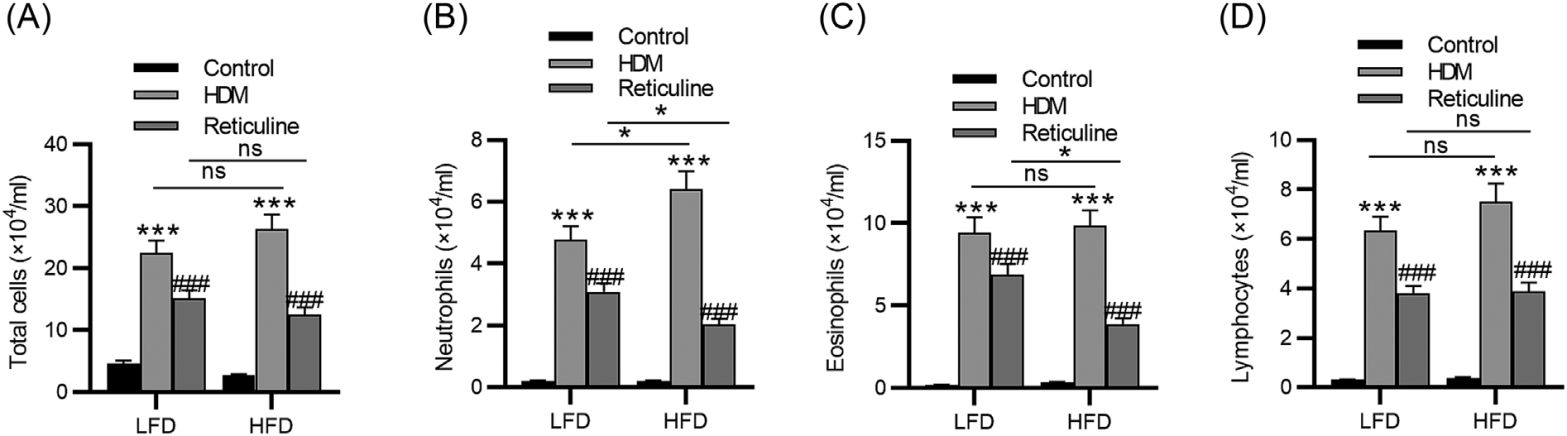
Reticuline suppresses the recruitment of inflammatory cells in BALF in obesity‐related asthma. (A) Number of total cells in BALF. (B) Number of neutrophils in BALF. (C) Number of eosinophils in BALF. (D) Number of lymphocytes in BALF. *n* = 6 per group. ***p < 0.001 compared to control, ^###^ *p* < 0.001 compared to HDM. HDM, house dust mite; LFD, low‐fat diet; HFD, high‐fat diet; BALF, bronchoalveolar lavage.

**FIGURE 3:**
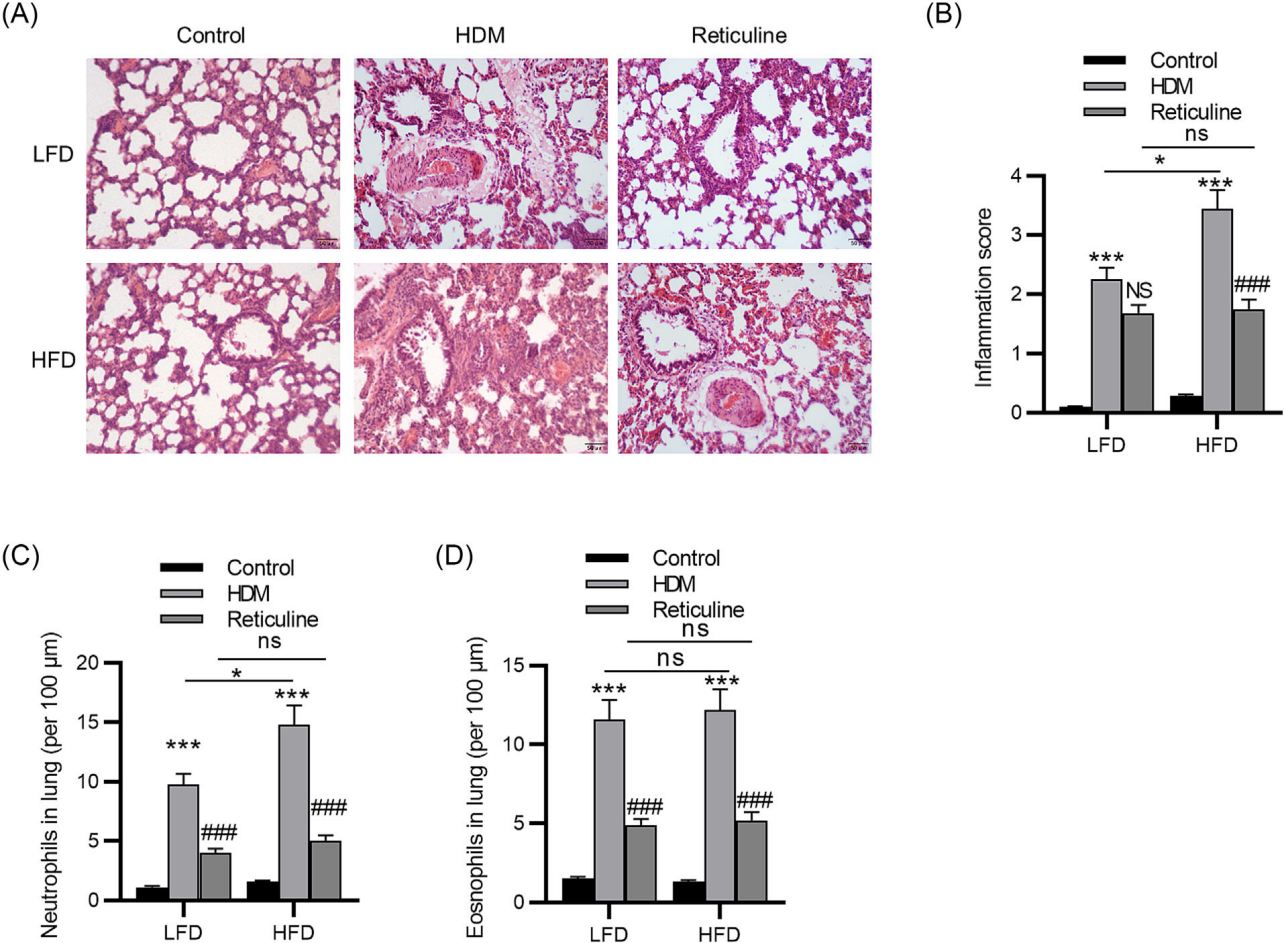
Reticulin attenuates lung inflammation in obesity‐related asthma. (A) Histological examination for airway inflammation. Sections were stained with H&E. (B) Slides were scored for peribronchial inflammation and airway mucosal hyperplasia, using a semiquantitative score from 0 to 4. (C) Quantification of neutrophil infiltration. (D) Quantification of eosinophil infiltration. *n* = 6 per group. ***p < 0.001 compared to control, ^###^ *p* < 0.001 compared to HDM. HDM, house dust mite; LFD, low‐fat diet; HFD, high‐fat diet; H&E, hematoxylin–eosin.

We investigated the effects of reticuline in mouse models of HDM‐induced airway inflammation with LFD or HFD. A substantial elevation in the number of total inflammatory cells, neutrophils, eosinophils, and lymphocytes in BALF was observed after HDM administration in both LFD and HFD groups. Additionally, among the HDM‐challenged mice, the number of neutrophils, not eosinophils and lymphocytes, was obviously increased in HFD mice compared to LFD mice (Figure 2A–D). However, reticuline treatment abolished these increases in both LFD and HFD groups. Lung histological examination was performed by H&E staining. The control group showed structurally normal tissue, with no or few inflammatory cells in the peribronchial and perivascular areas, while the infiltration of inflammatory cells into the connective tissue surrounding the bronchial and bronchiolar segments was observed in asthma mice. Notably, asthma mice fed HFD exhibited more obvious inflammatory cell infiltration than LFD‐fed asthma mice. However, the inflammation in obesity‐related mice was ameliorated by reticuline in the HFD group (Figure 3A,B). The reticuline treated group showed significantly reduced neutrophil and eosinophils infiltration than the untreated group (Figure 3C,D). Additionally, the same results were obtained from the HFD and OVA‐induced obesity‐related asthma models (Figure S1B–D).

### 3.3 Reticuline reduces proinflammatory cytokine levels in obesity‐related asthma

**FIGURE 4:**
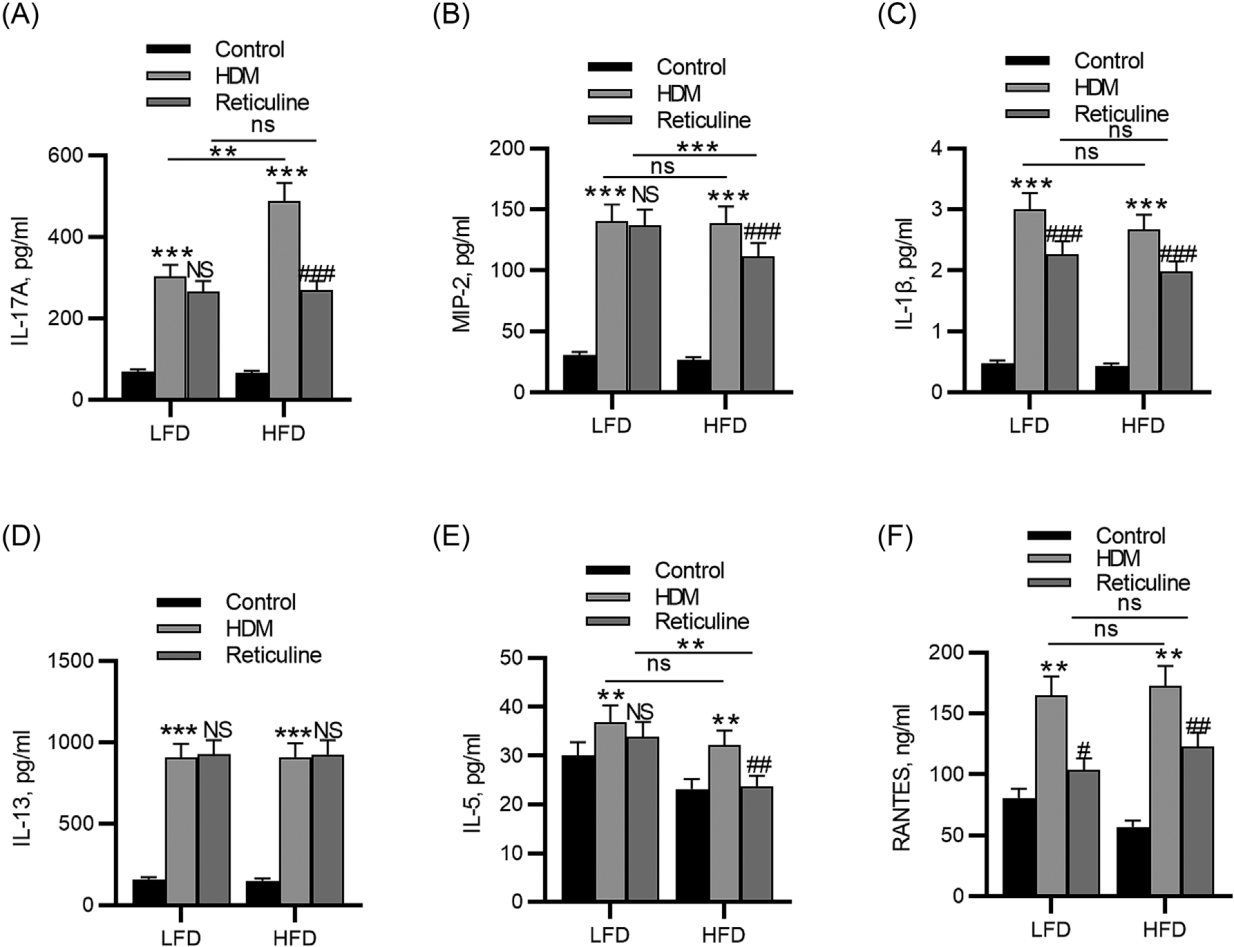
Reticuline reduces proinflammatory cytokine levels in obesity‐related asthma. (A) Concentration of IL‐17A. (B) MIP‐2. (C) IL‐1β. (D) IL‐13. (E) IL‐5. (F) RANTES in lung tissues were measured by ELISA kits. *n* = 6 per group. **p < 0.01, ***p < 0.001 compared to control, ^#^ *p* < 0.05, ^##^ *p* < 0.01, ^###^ *p* < 0.001 compared to HDM. HDM, house dust mite; LFD, low‐fat diet; HFD, high‐fat diet; IL‐17A, interleukin‐17A; MIP‐2, macrophage inflammatory protein 2; IL‐1β, interleukin‐1β; IL‐13, interleukin‐13; IL‐5, interleukin‐5; RANTES, regulated on activation, normal T cell expressed and secreted.

The levels of cytokines in the lung were determined using ELISA. The results demonstrated that HDM administration significantly upregulated the concentrations of IL‐17A, MIP‐2, IL‐1β, IL‐13, IL‐5, and RANTES in both LFD and HFD groups. Administration of reticuline counteracted the effects of HDM on IL‐17A, MIP‐2, IL‐1β, IL‐5, and RANTES levels in obese mice, and IL‐13 the level was not affected by reticuline (Figure 4A–F). The concentrations of IL‐1β and RANTES were reduced by reticuline in the LFD‐HDM group, suggesting that reticuline suppressed both type 2‐low neutrophilic airway inflammation and type 2 eosinophilic airway inflammation.

### 3.4 Reticuline inactivates the JAK2/STAT3/SOCS3 and p38 MAPK/NF‐κB signaling pathways

**FIGURE 5:**
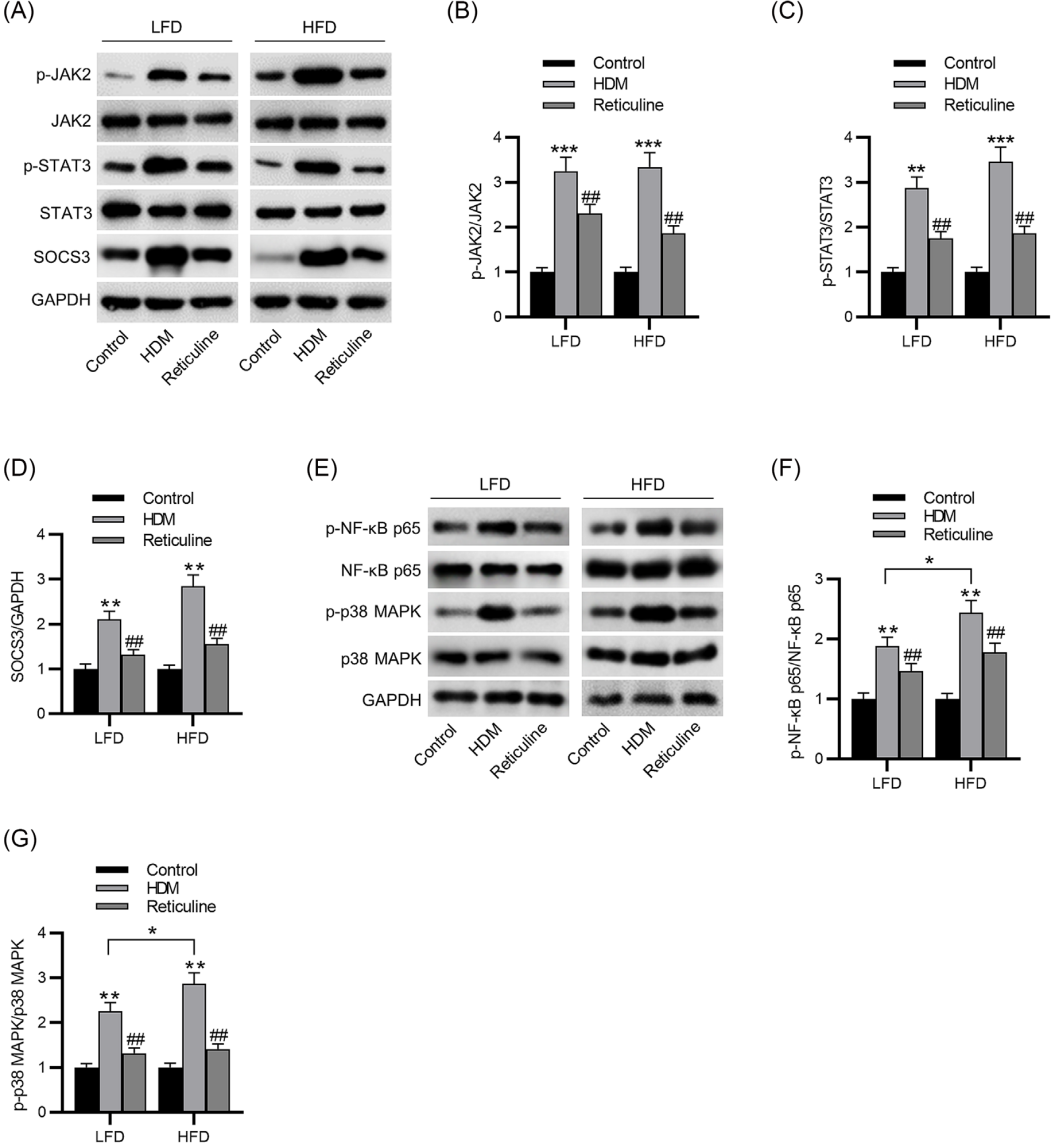
Reticuline inactivates the JAK2/STAT3/SOCS3 and p38 MAPK/NF‐κB signaling pathways. (A–D) Western blotting analysis and quantification of p‐JAK2, JAK2, p‐STAT3, STAT3, and SOCS3 in lung tissues. (E–G) Western blotting analysis and quantification of p‐NF‐κB p65, NF‐κB p65, p‐p38 MAPK, and p38 MAPK in lung tissues. *n* = 6 per group. **p < 0.01, ***p < 0.001 compared to control, ^##^ *p* < 0.01 compared to HDM. JAK2, Janus kinase 2; STAT3, signal transducer and activator of transcription 3; SOCS3, suppression of cytokine signaling 3; NF‐κB, nuclear factor kappa B; MAPK, mitogen‐activated protein kinase.

Mechanistically, the results of western blotting suggested that reticuline inhibited the HDM‐induced upregulation in the phosphorylated levels of JAK2 and STAT3 and the levels of SOCS3 in both LFD and HFD groups (Figure 5A–D), suggesting that reticuline blocked the JAK2/STAT3/SOCS3 signaling in obesity‐related asthma mice. Interestingly, the immunoblotting results demonstrated that the phosphorylated levels of NF‐κB p65 and p38 MAPK were increased in asthma mice and obesity‐related asthma mice compared to control. Additionally, in the asthma groups, their phosphorylated levels were upregulated by HFD compared to LFD. However, reticuline markedly inhibited the activation of p38 MAPK/NF‐κB signaling pathways in asthma mice and obesity‐related asthma mice (Figure 5E–G). Additionally, we further demonstrated the inhibitory effects of reticuline on the JAK2/STAT3/SOCS3 and p38 MAPK/NF‐κB signaling pathways in the HFD and OVA‐induced obesity‐related asthma models (Figure S2A–G).

## 4 DISCUSSION

Obese patients with asthma are insensitive to medical treatment. Here, an experimental model of obesity‐related asthma was established. The current study provided the first evidence to show the role of adiponectin and its mechanism in obesity‐related asthma. We found that the IgE level was significantly elevated in asthma mice and obesity‐related asthma mice, which indicated a mouse model of obesity‐related allergic asthma was well‐established. Airway resistance and airway inflammation were exacerbated in obesity‐related asthma mice compared to asthma mice. These findings provided the support for the suggestion that obesity is an independent risk factor for asthma. The histological examination results showed that reticuline reduces the inflammatory cell recruitment in lung tissues of obese mice with HDM or OVA‐induced obesity‐related asthma. Airway hyperreactivity is a typical clinical symptom of asthma, which is characterized by abnormal increase in airflow limitation in response to a provoking stimulus.^31^ Here, we found that reticuline notably attenuated airway resistance in asthma mice and obesity‐related asthma mice induced by HDM or OVA.

Asthma is divided into Th2‐high (eosinophilic airway inflammation) and Th2‐low asthma (neutrophilic asthma). In addition to airway inflammation, inflammation‐independent process also induces asthma initiation.^32^, ^33^ Yang et al. suggest that reticuline reduces the infiltration of neutrophil leukocytes in rat paw tissue.^18^ Here, reticuline decreased the number of total inflammatory cells, neutrophils, and eosinophils in BALF in both LFD‐ and HFD‐fed asthmatic mice. Circulating neutrophils are activated in severely obese asthma,^34^ and this previous finding is consistent with the current data that HFD increased the risk of neutrophilic airway inflammation, rather than eosinophilic airway inflammation. Moreover, reticuline treatment significantly decreases the production of TNF‐α and IL‐6 in foot tissue homogenates.^18^ An increase in the IL‐17A level is associated with neutrophilia in obese asthmatic patients.^35^ Macrophages can infiltrate adipose tissues and regulate local metabolism.^36^ Here, administration of reticuline decreased the production of IL‐17A, MIP‐2, IL‐1β, IL‐5, and RANTES in lung tissues of mice with obesity‐related asthma induced by HDM or OVA. Taken together, reticuline might repress airway inflammation in obesity‐related asthma via preventing the recruitment of inflammatory cells and inflammatory cytokines.

We focused on several intracellular pathways involved in the control of allergic inflammation. JAK2 phosphorylation activates STAT3 and SOCS3 release. The role of STAT3 in asthma is still controversial, as some reports have indicated that the STAT3 level is elevated in asthma, while others have found that blocking this signaling pathway improves asthmatic symptoms. Suppression of STAT3 prevents the production of IL‐4, IL‐13, and IL‐17, which are upregulated in asthma.^37^ An upregulation in the phosphorylated JAK2 and STAT3 and SOCS3 protein levels were detected in lung tissues of asthmatic mice in this study. Treatment with reticuline reduced this response. p38 is a key component of the MAPK pathway, required for triggering the inflammatory response the regulation of IL‐13 and RANTES.^38^, ^39^ Intriguingly, the RANTES level in obesity‐related asthma induced by HDM or OVA was attenuated by reticuline, which might be related to the reduction in p38 phosphorylation by reticuline. Obesity patients are in a state of chronic inflammation.^40^ Upregulation of inflammatory cytokines in obesity patients may activate the NF‐κB signaling. In obese mouse models, activation of NF‐κB signaling fed HFD for 12 weeks is approximately 3.5‐fold than that of the baseline level.^41^ Furthermore, the cytokines regulated by NF‐κB, including IL‐1β, IL‐2, ICAM‐1, and VCAM‐1 are associated with obese asthma.^42^ In this study, we demonstrated that reticuline reduced the levels of NF‐κB p65 phosphorylation to block NF‐κB signaling in mice with obesity‐related asthma induced by HDM or OVA.

Sensitization methods utilizing HDM and OVA are known to enhance manifestations of asthmatic features. HDM‐ and OVA‐induced models are considered the appropriate methods for experimental allergic asthma. These models have similar clinical symptoms to human asthma, which are characterized by airway inflammation, mucus hypersecretion, thickening of bronchial wall, and increased infiltration of inflammatory cells into the lung.^43^ HDM has become more commonly used in mouse models to induce AHR because of its immunogenic properties, so the use of an adjuvant is not required.^44^ In this study, reticuline was administrated into OVA‐ and HDM‐induced asthmatic models. Our data showed that reticuline treatment produced a similar protective effect in lean and obese asthmatic mice. The findings of the two‐way analysis of variance indicated that baseline airway resistance, the number of neutrophils, inflammation score, and IL‐17A levels were notably elevated in obesity‐related asthma mice (HDM‐induced models) compared to lean asthma mice. Interestingly, reticuline treatment significantly ameliorated these changes. Furthermore, the number of neutrophils and inflammation score were increased in obesity‐related asthma mice (OVA‐induced models) compared to lean asthma mice. Reticuline treatment also ameliorated these changes induced by OVA. These findings indicated this drug served both conditions. Importantly, there was no significant difference between the reticuline treated obese group and the reticuline treated lean group, suggesting the efficacy of reticuline in treating obesity‐related asthma.

In conclusion, this study showed that reticuline attenuated airway inflammation in a mouse model of obesity‐associated asthma. Clinical use of reticuline might be promising because reticuline has therapeutic effects on asthma with obesity. In the further studies, we will look at the cell counts in bone marrow (and circulating blood) to investigate whether reticuline presents an impaired immunosuppressive effect, which will provide more evidence for the pharmacologic action of reticuline.

## AUTHOR CONTRIBUTIONS

Xiaojiang Lyu and Jiaojiao Liu conceived and designed the experiments. Xiaojiang Lyu, Jiaojiao Liu, Zengrong Liu, Ying Wu, Ping Zhu, and Chonghai Liu carried out the experiments. Xiaojiang Lyu, Jiaojiao Liu, and Chonghai Liu analyzed the data. Xiaojiang Lyu, Jiaojiao Liu, and Chonghai Liu drafted the manuscript. All authors agreed to be accountable for all aspects of the work. All authors have read and approved the final manuscript.

## CONFLICT OF INTEREST STATEMENT

The authors declare that they have no competing interests.

## ETHICS STATEMENT

Animal care and experimental protocols were consistent with the Guide for the Care and Use of Laboratory Animals and approved by the Ethics Committee of Affiliated Hospital of North Sichuan Medical College (2022ER512‐1).

## Supporting information


**Figure S1.** **Reticulin attenuates airway inflammation in obesity‐related asthma induced by OVA.** (A) Protocol for OVA‐induced airway inflammation treated with reticuline in mice receiving LFD or HFD. (B) Histological examination for airway inflammation. Sections were stained with H&E. (C) Slides were scored for peribronchial inflammation and airway mucosal hyperplasia, using a semiquantitative score from 0 to 4. (D) Number of eosinophils in BALF. n = 6 per group. ^**^ *p* < 0.01 compared to control, ^##^ *p* < 0.01 compared to OVA. OVA, ovalbumin; LFD, low‐fat diet; HFD, high‐fat diet; H&E, hematoxylin–eosin.

**Figure S2.** **Reticuline inactivates the JAK2/STAT3/SOCS3 and p38 MAPK/NF‐κB signaling pathways in obesity‐related asthma induced by OVA.** (A‐D) Western blotting analysis and quantification of p‐JAK2, JAK2, p‐STAT3, STAT3, and SOCS3 in lung tissues. (E‐G) Western blotting analysis and quantification of p‐NF‐κB p65, NF‐κB p65, p‐p38 MAPK, and p38 MAPK in lung tissues. n = 6 per group. ^**^ *p* < 0.01 compared to control, ^##^ *p* < 0.01 compared to OVA. JAK2, Janus kinase 2; STAT3, signal transducer and activator of transcription 3; SOCS3, suppression of cytokine signaling 3; NF‐κB, nuclear factor kappa B; MAPK, mitogen‐activated protein kinase.

## Data Availability

The datasets used or analyzed during the current study are available from the corresponding author on reasonable request.
