# Supplementary figures and images for: Anti‐inflammatory effects of reticuline on the JAK2/STAT3/SOCS3 and p38 MAPK/NF‐κB signaling pathway in a mouse model of obesity‐associated asthma
Source: Clin Respir J. 2024 Jan 19;18(1):e13729. doi: 10.1111/crj.13729 (PMC10799233; doi:10.1111/crj.13729)

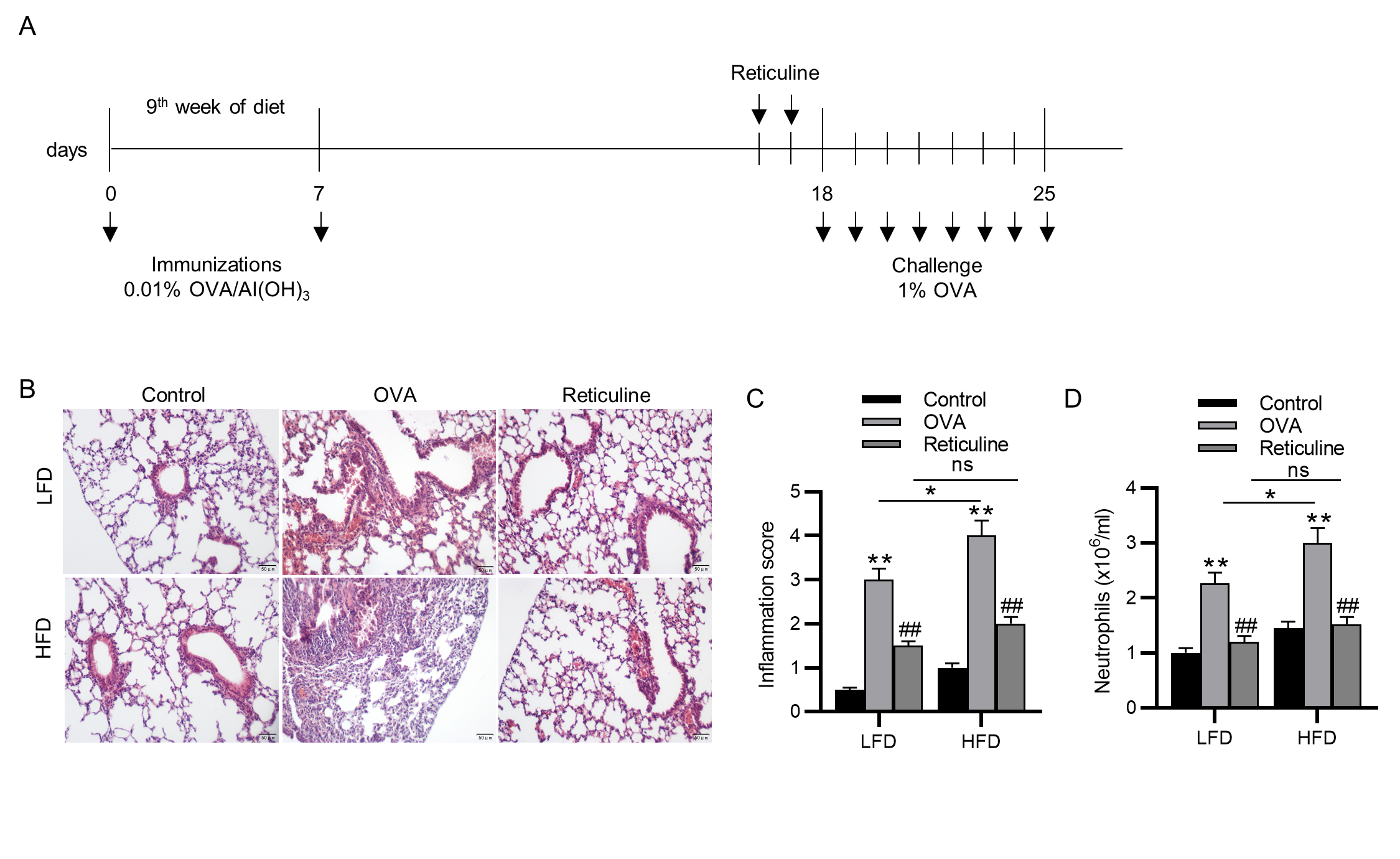

Supplement: Supplementary file 1 — Figure S1. Reticulin attenuates airway inflammation in obesity‐related asthma induced by OVA. (A) Protocol for OVA‐induced airway inflammation treated with reticuline in mice receiving LFD or HFD. (B) Histological examination for airway inflammation. Sections were stained with H&E. (C) Slides were scored for peribronchial inflammation and airway mucosal hyperplasia, using a semiquantitative score from 0 to 4. (D) Number of eosinophils in BALF. n = 6 per group. ** p < 0.01 compared to control, ## p < 0.01 compared to OVA. OVA, ovalbumin; LFD, low‐fat diet; HFD, high‐fat diet; H&E, hematoxylin–eosin. [file CRJ-18-e13729-s002.tif]

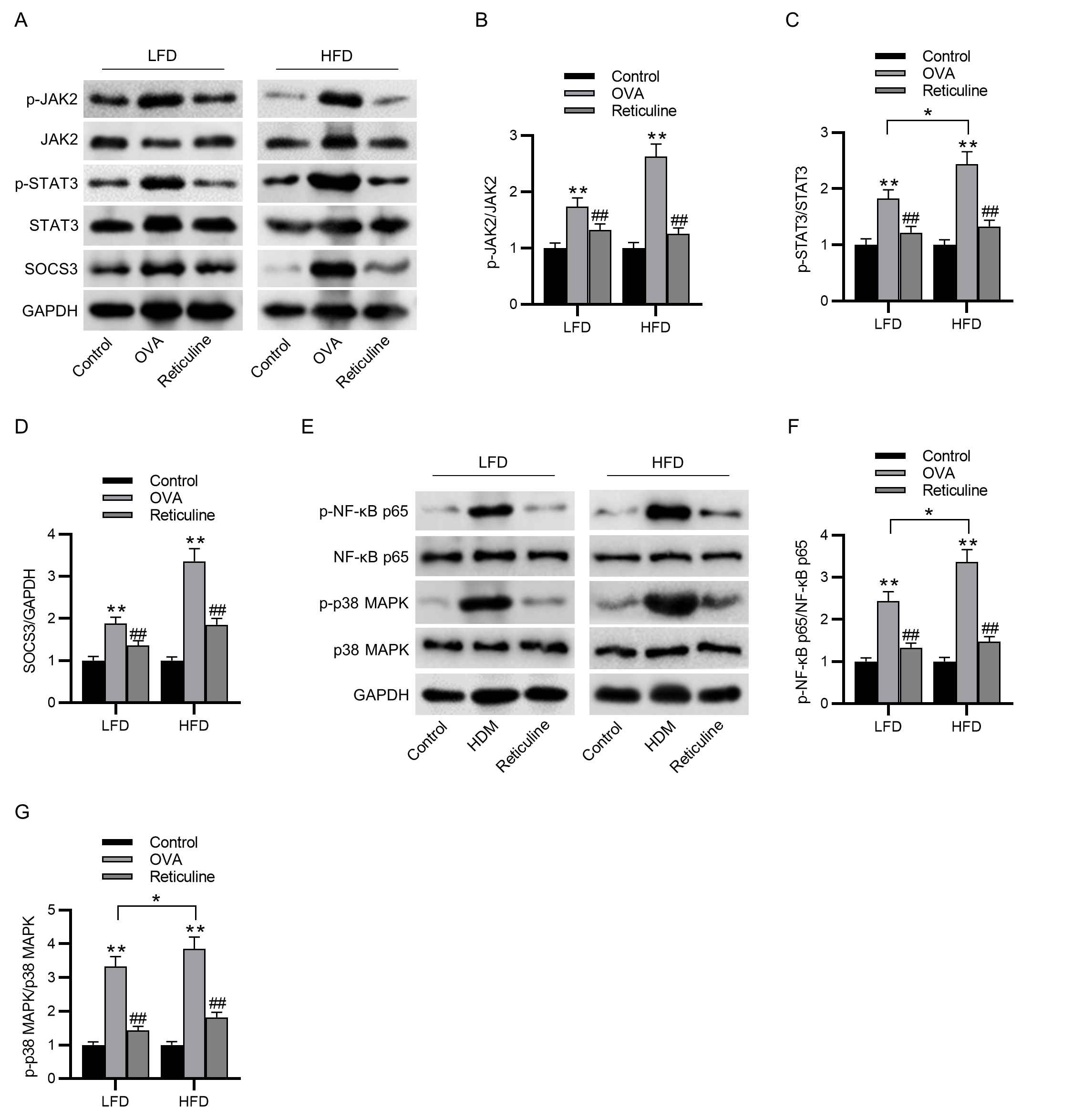

Supplement: Supplementary file 2 — Figure S2. Reticuline inactivates the JAK2/STAT3/SOCS3 and p38 MAPK/NF‐κB signaling pathways in obesity‐related asthma induced by OVA. (A‐D) Western blotting analysis and quantification of p‐JAK2, JAK2, p‐STAT3, STAT3, and SOCS3 in lung tissues. (E‐G) Western blotting analysis and quantification of p‐NF‐κB p65, NF‐κB p65, p‐p38 MAPK, and p38 MAPK in lung tissues. n = 6 per group. ** p < 0.01 compared to control, ## p < 0.01 compared to OVA. JAK2, Janus kinase 2; STAT3, signal transducer and activator of transcription 3; SOCS3, suppression of cytokine signaling 3; NF‐κB, nuclear factor kappa B; MAPK, mitogen‐activated protein kinase. [file CRJ-18-e13729-s001.tif]
